# Social isolation during COVID‐19 lockdown impairs cognitive function

**DOI:** 10.1002/acp.3821

**Published:** 2021-03-24

**Authors:** Joanne Ingram, Christopher J. Hand, Greg Maciejewski

**Affiliations:** ^1^ School of Education and Social Science University of the West of Scotland Paisley UK; ^2^ Department of Psychology Glasgow Caledonian University Glasgow UK

**Keywords:** cognitive decline, COVID‐19, executive function, lockdown, social isolation

## Abstract

Studies examining the effect of social isolation on cognitive function typically involve older adults and/or specialist groups (e.g., expeditions). We considered the effects of COVID‐19‐induced social isolation on cognitive function within a representative sample of the general population. We additionally considered how participants ‘shielding’ due to underlying health complications, or living alone, performed. We predicted that performance would be poorest under strictest, most‐isolating conditions. At five timepoints over 13 weeks, participants (*N* = 342; aged 18–72 years) completed online tasks measuring attention, memory, decision‐making, time‐estimation, and learning. Participants indicated their mood as ‘lockdown’ was eased. Performance typically improved as opportunities for social contact increased. Interactions between participant sub‐groups and timepoint demonstrated that performance was shaped by individuals' social isolation levels. Social isolation is linked to cognitive decline in the absence of ageing covariates. The impact of social isolation on cognitive function should be considered when implementing prolonged pandemic‐related restrictive conditions.

## 1 INTRODUCTION

Much of the global population has experienced ‘lockdown’ conditions due to the COVID‐19 pandemic. There is growing evidence of the consequences of COVID‐19‐related social isolation, confinement, and loneliness on mood and physical health (Lippi et al., 2020; Zhang et al., 2020), but no examination of similar changes in cognitive function has been presented. If ‘lockdown’ conditions lead to cognitive decline—in memory, perceptual ability, and/or executive function—this has broad impact for education, work, and everyday life, as well as implications for theories of cognitive decline.

COVID‐19 restrictions vary from country‐to‐country and vary across time within countries. In Scotland, the strictest conditions permitted leaving home only for societally essential work, groceries, and solo exercise once a day (or exercise with members of one's own household). Entering another home was only permitted in emergencies. Additionally, approximately 1‐in‐20 adults were required to ‘shield’ due to pre‐existing conditions which made them vulnerable to COVID‐19 infection/complications. Shielding individuals were required to stay at home, indoors at all times, initially with no exceptions. Effectively, citizens were left entirely isolated (if living alone) or were restricted to interpersonal contact with only members of their household.

Except for outdoor exercise, which became unlimited after 49 days, residents of Scotland spent 66 days under the strictest lockdown conditions. Severe and suddenly imposed constraints on interpersonal contact led, for some, to feelings of isolation and loneliness (Li & Wang, 2020), and to higher levels of negative mood (Ingram et al., 2020). The relationship between isolation and cognitive decline in certain populations has been well‐documented (see Cacioppo & Hawkley, 2009 for a review); we investigated if isolation due to COVID‐19 restrictions led to a decline in cognitive function in the general population, with specific consideration of those shielding and/or living alone.

Social isolation and cognitive decline are typically assessed in older adults. Findings are frequently inconsistent as measurement of social activity is variable (Evans, Martyr, et al., 2019), and social isolation is difficult to rigorously control. However, isolation has been shown to influence cognitive functioning (Evans, Martyr, et al., 2019) and decline (Kuiper et al., 2016). Living alone and having no close relationships, or having a limited or poor social network have been linked to increased risk of dementia (Fratiglioni et al., 2000), whilst poorer cognitive ability in the absence of dementia has been predicted by lower levels of emotional support (Seeman et al., 2001). Social engagement during recreational activities enhances memory (Richards et al., 2003), and protects against cognitive decline (Bassuk et al., 1999). Younger participants who were experimentally induced to envisage a future of social isolation were impaired on general mental ability, self‐regulation, and reasoning (Baumeister et al., 2005; Baumeister & DeWall, 2005; Twenge et al., 2001). Recent research suggests that when controlling for age, gender, education level, and physically limiting health conditions, social isolation (the absence of social relationships and disengagement from community; Nicholson, 2009) is associated with level of cognitive function (Evans et al., 2018; Evans et al., 2019).

Associations between social isolation and cognitive function are frequently linked to cognitive reserve (Stern, 2009). Social interactions with others involve mental stimulation, hence frequent social interaction may protect or enhance cognitive function (van Gelder et al., 2006). Reserve and maintenance of cognitive function may be protected through regular effortful social interactions which require engagement of complex cognitive processes (Barnes et al., 2004; Fratiglioni et al., 2004). However, research in this area is limited due to the inability to establish the directional link between cognitive and social decline—that is, those experiencing greater decline may be unable to maintain social interactions. Studies have generally controlled for this using baseline measurements (Barnes et al., 2004; Fratiglioni et al., 2004; Zunzunegui et al., 2003) and existing cohort data (Gow et al., 2007). However, research has indicated causal links using cross‐lagged modelling (Thomas, 2011) and latent change score modelling (Read et al., 2020). These studies found differential effects of social isolation on cognitive impairment across males and females. Whilst neurological and situational ageing effects are impossible to accurately imitate, enforced lockdown conditions afforded a unique opportunity to replicate certain social and physical restrictions often experienced only by older adults. Further issues with measurement of social contacts and networks (Evans, Martyr, et al., 2019) were mitigated by the blanket rule of no‐contact across the region.

Social isolation effects have also been assessed naturalistically during scientific expeditions. A study of prolonged Antarctic isolation yielded varied results, with clear functional detriment only evident at the very end of the isolation period (Khandelwal et al., 2017). However, while isolated from broader society, that expedition facility housed 26 team‐members, allowing for extensive, varied face‐to‐face interpersonal contact. Examination of a solitary participant during a 17‐day expedition on‐foot through the Simpson desert indicated substantial cognitive deterioration over time, which resolved fully once the expedition was complete (Maruff et al., 2006).

A review of Antarctic expeditions (Zimmer et al., 2013) noted that 63.6% of studies reported cognitive impairment, with a variety of aetiologies suggested, including stress and fatigue, and low environmental stimulation. However, other studies (John Paul et al., 2010; Palinkas et al., 2005) demonstrate maintained or even improved cognitive performance over extended periods in polar environments. Studies of spaceflight have yielded mixed evidence; detriment is typically attributed to effects of microgravity or environmental stressors as opposed to social isolation (Kanas & Manzey, 2008). Deficits in attentional processing (Pattyn et al., 2005) and concurrent task‐management (Manzey & Lorenz, 1998) have been found, but individual effects of social isolation or stressful environment are rarely demonstrated. Collectively, the results of studies on the effects of social isolation on cognitive function during expeditions show mixed results or no detriment to cognition. However, it is important to note that astronauts and polar explorers are carefully selected against specific criteria and undergo rigorous medical and psychological screening (De La Torre et al., 2012; John Paul et al., 2010). Space expeditions are generally short, allowing little time to experience effects of isolation. Polar expeditions often involve a larger number of individuals, which perhaps provides sufficient social contact to maintain function. Finally, these individuals have consented to enter a restrictive environment; thus, these groups/findings may not be representative when considering the effects of pandemic‐related social isolation.

Disentangling the effect of social isolation on human cognitive function is difficult, but we can draw parallels with animal studies. Rats reared in isolation demonstrate deficits in cognitive flexibility (Amitai et al., 2014); isolating animals impairs reversal learning, regardless of inanimate stimulation, suggesting isolation effects on prefrontal cortico‐striatal pathways (Schrijver et al., 2004). Studies further demonstrate that social isolation leads to permanent neurochemical, behavioural, and neurostructural changes in rodents (Jones et al., 2011; Schubert et al., 2009).

Research involving older adults or expeditions suggest that cognitive function can be improved or restored through cognitive plasticity. Research on plasticity in older adults ties closely with the notion of cognitive reserve already discussed (see Hertzog et al., 2009 for a discussion of cognitive enrichment). Studies have shown that with cognitive and/or physical training or intervention, cognitive function can be maintained or improved in the ageing brain (Bherer, 2015; Karbach & Verhaeghen, 2014). Cognitive decline seen in expeditioners has been found to resolve quickly after the expedition was complete (Maruff et al., 2006; Ratino et al., 1988), suggesting that short‐term periods of isolation do not impact cognitive function over the longer‐term. Considering the evidence for plasticity of cognitive function, it was expected that any cognitive decline resulting from COVID‐19 restrictions to social contact would resolve as restrictions were relaxed.

Societal ‘lockdown’ conditions within the UK (beginning in March 2020) provided a valuable opportunity to assess social isolation effects on cognitive function across a large representative sample, with minimal limitations (e.g., older‐adult‐only sample, extreme environments). To compare cognitive function during stricter and more‐liberal societal conditions (i.e., when extra‐household face‐to‐face social contact was permitted), participants completed multiple cognitive tasks across five timepoints. Tasks assessed a range of cognitive functions, examined previously in relation to social isolation (Benke et al., 1993; John Paul et al., 2010; Kelly et al., 2005; Ratino et al., 1988). These included: attention (Flanker Task: Wylie et al., 2007), working memory (Digitized‐Digit Symbol Substitution Task; Chatterjee et al., 2019), decision making (Iowa Gambling Task, Bechara et al., 1994), time perception, (modified version of Time Production Task; Tortello et al., 2020) and learning (Symbol Learning; Yang et al., 2017).

The initial timepoint (Week 1) aligned with participants living under the most‐restrictive conditions—leaving the house was allowed only for non‐shielding individuals for essential work which could not be completed from home, for essential groceries, or for individual outdoor exercise (which had become unlimited, after initially being restricted to once per day). Participants completed the task battery at four further timepoints. Restrictions were eased across this period as follows. At Week 3, unless self‐isolating/shielding, meeting outside with one other household was allowed. At Week 5, one household could meet with people from up to two households out‐of‐doors, those in the shielding group could go outdoors for exercise. At Week 9, people could meet with others from up to two households indoors or outdoors, and retail, hospitality, hairdressers, and cultural venues re‐opened. At Week 13, in addition to the expansion at Week 9, children had returned to nurseries and schools.

We predicted that performance on all tasks would be poorest at timepoint 1, with gradual improvement as restrictions were eased. We predicted that due to differing levels of isolation, shielding participants would show differential effects to non‐shielding participants, and that those who lived alone would show differential effects to those who co‐habited.

## 2 METHOD

### 2.1 Participants

Three hundred forty‐two Scottish nationals/long‐term residents (56.7% female, 41.5% male, 0.6% non‐binary, 0.9% transgender) aged 18–72 years old (*M*_age_ = 32.1 years, *SD*_age_ = 11.2) participated. An a priori power analysis anticipating small effect sizes (*f* = 0.10, *α* = 0.05, power = 0.95) suggested a target sample of 188; thus, our sample was ample. Participants who identified as Scottish were recruited using Prolific Academic (https://prolific.co) and first took part in an additional study on the effects of COVID‐19 restrictions on healthy behaviours (Ingram et al., 2020). Three hundred ninety‐nine eligible participants took part in the additional study by Ingram et al. Participants who were native English‐speakers with no vision/attention/learning impairment or prior knowledge of Mandarin characters (used in the symbol‐learning task; *N* = 342), were then immediately invited to take part in the current study. A breakdown of key participant demographic characteristics is presented in Table 1. There are two rows of data in Table 1; one represents the main dataset, and the other represents the subset dataset (please see Section 2.3 for further details). Crucially, there are very few differences in the demographic make‐up of the sub‐sample, relative to the global sample.

**TABLE 1:** Participant sample demographics

*Sample*	*N*	*Mean age*				
Main	342	32.1 years (*SD* = 11.2)				
Subset	203	33.4 years (*SD* = 11.9)				
*Gender‐Sex*	*Female*	*Male*	*Non‐binary*	*Trans*		
Main	56.6%	41.3%	0.9%	0.6%		
Subset	56.2%	42.4%	0.5%	1.0%		
*Location*	*Town*	*City*	*Suburbs*	*Village*	*Countryside*	
Main	32.2%	26.6%	22.2%	12.6%	6.4%	
Subset	29.6%	27.1%	24.6%	10.8%	7.9%	
*Relationship status*	*Single*	*Married*	*In a relationship*	*Divorced*	*Separated*	
Main	28.9%	26.6%	41.8%	0.9%	1.8%	
Subset	30.1%	25.1%	42.9%	1.0%	1.0%	
*Household*	*Partner only*	*Parents*	*Partner + Children*	*Living alone*	*Other adult*	*Alone + child(ren)*
Main	29.2%	24.9%	21.4%	12.0%	6.4%	3.5%
Subset	24.9%	30.0%	20.8%	16.2%	5.1%	3.1%
*Student status*	*Full‐time*	*Part‐time*	*Non‐student*			
Main	22.0%	3.5%	74.5%			
Subset	18.2%	3.5%	78.3%			
*Employment*	*Working from home*	*Unemployed*	*Furloughed*	*Keyworker*	*Carer/parent*	*Working away*
Main	36.1%	19.9%	21.1%	14.4%	4.7%	2.3%
Subset	35.9%	21.2%	20.7%	13.6%	6.1%	2.5%
*Physical activity*	*A lot less active*	*A little less active*	*About the same*	*A little more active*	*A lot more active*	
Main	23.8%	22.0%	15.8%	25.8%	12.6%	
Subset	22.7%	25.1%	15.3%	22.7%	14.3%	

All experienced social isolation during lockdown; 14.9% (*n*_shield_ = 51) of participants identified as having ‘shielded’ throughout lockdown. Approximately 3% of the general Scottish adult population were ‘required’ to shield (Scottish Government, 2020). 12% of participants lived alone during lockdown—in the broader Scottish context approximately 15% of people live alone (National Records Scotland, 2018). There was moderate participant dropout across timepoints (328 participants remained after Week 3, 275 after Week 5, 228 after Week 9, and 203 after Week 13). No participants were excluded during the study. A small number of participants were not included in certain sub‐analyses; specific details can be found under Section 2.3.

### 2.2 Measures and procedure

We examined participants' performance on five cognitive tasks. These included the Iowa gambling task (adapted from Bechara et al., 1994) as a measure of decision making, a flanker task (adapted from Wylie et al., 2007) as a measure of selective attention, a symbol‐learning task (adapted from Yang et al., 2017) as a measure of learning ability, a digit‐symbol substitution task (Chatterjee et al., 2019, Version 1) as a measure of working memory, and a time production task (adapted from Tortello et al., 2020) as a measure of time estimation. As negative mood has been shown to correlate with poorer performance on some cognitive tasks (see Chepenik et al., 2007 for a review), we measured and controlled for participants' negative mood when examining potential changes in cognitive function. Ten negative items from Grove and Prapavessis' (1992) abbreviated Profile of Mood State (POMS) scale were used. For more information about the tasks and measures, please see the Supporting Information.

The tasks were designed and administered online, using the Gorilla Experiment Builder (https://gorilla.sc; for information about stimulus and response timing precision, see Anwyl‐Irvine et al., 2020; Bridges et al., 2020). The tasks were administered in the same order at each timepoint (Iowa gambling, flanker, symbol‐learning, time production, digit‐symbol substitution, mood rating). At the end of timepoint 5 (Week 13), could disclose whether they had cheated on any of the tasks (e.g., using an online translator to determine Mandarin character meanings). Participants received £5 for completing each session; sessions took, on average, 20 min to complete. The study was approved by the lead institution ethics committee, following British Psychological Society (2014) guidelines.

### 2.3 Data analysis

Cognitive task data were analysed with four logit/linear mixed‐effects models, using the ‘lme4’ package (Bates et al., 2011) in *R* (R Development Core Team, 2004). Fixed effects were tested using maximum likelihood‐ratio tests comparing full and reduced models. The first model (henceforth referred to as the ‘main’ model) included Time (Weeks 1, 3, 5, 9, 13) as a fixed effect and Negative Mood Rating (NMR), Age, and Gender as covariates. Within Time, repeated coding was used to define four planned contrasts that compared consecutive timepoint pairings (Week 1 vs. 3, Week 3 vs. 5, Week 5 vs. 9, Week 9 vs. 13). Note that NMR was removed due to model non‐convergence^1^ for the Iowa gambling, flanker (accuracy but not RT), symbol‐learning, and digit‐symbol substitution (accuracy) tasks. For the same reason, Age was removed for the Iowa gambling, symbol‐learning (only analyses involving interaction terms), and digit‐symbol substitution tasks (accuracy), whilst Gender was removed for the Iowa gambling (only analyses involving interaction terms), flanker (accuracy), symbol‐learning, and digit‐symbol substitution (accuracy) tasks. NMR and Age were significant covariates of RT in the flanker and digit‐symbol substitution tasks. Age was also a significant covariate of accuracy in the digit‐symbol substitution task. Gender was a significant covariate of RT in the flanker and DSST tasks and the number of advantageous deck selections in the Iowa gambling task.

The second model (henceforth referred to as the ‘subset’ model) was identical to the main model, except that it included only those participants who completed all sessions (203 out of 342). The rationale for a second model was that due to participant dropout, estimates for each timepoint in the main model could be biased as they were based on all participants who completed a given session, rather than those who completed all sessions. This is because mixed‐effects models ignore missing observations (unlike general linear models which delete them listwise). However, we demonstrate below that for each task, the results of the subset model corroborated those of the main model, confirming that the latter were not driven by a subset of participants at a particular timepoint.

The third model (henceforth referred to as the ‘shielding status’ model) examined differences between shielding (*n* = 51) and non‐shielding participants (*n* = 288). Note that three participants were excluded from these analyses as they did not disclose their shielding status. The fourth model (henceforth referred to as the ‘living status’ model) examined differences between participants who lived alone during lockdown (*n* = 41) and those who co‐habited (*n* = 301). The shielding status and living status models were identical to the main model, but additionally included Group (shielding, non‐shielding/solitary, non‐solitary) and Group × Time as additional fixed effects. For significant interactions, follow‐up comparisons examined Group differences for each pair of consecutive timepoints separately. The purpose of Supplementary by‐groups models was to demonstrate that the predicted gradual improvement in task performance was due to the easing of lockdown restrictions and differential social isolation, rather than due to simple practice effects.

NMR data were analysed using a linear mixed‐effects model with Time as a fixed effect. All models included a random intercept by‐participants. The random by‐participants slope for Time was significant in all models but was removed due to model non‐convergence after fixed effects and covariates were added.

Two of the 342 participants were excluded from the symbol‐learning task analyses; although they did not understand Mandarin, they reported knowing certain characters because they are also used in Japanese Kanji. Finally, RT analyses of flanker and digit‐symbol substitution tasks excluded incorrect responses (3.0% and 2.2% of responses, respectively), and excluded correct responses ±2 *SD*s from a participant's mean at each timepoint (4.0% and 4.7% of correct responses, respectively).

All data and analysis scripts are openly available (Ingram et al., 2021).

## 3 RESULTS

### 3.1 Iowa gambling task

**FIGURE 1:**
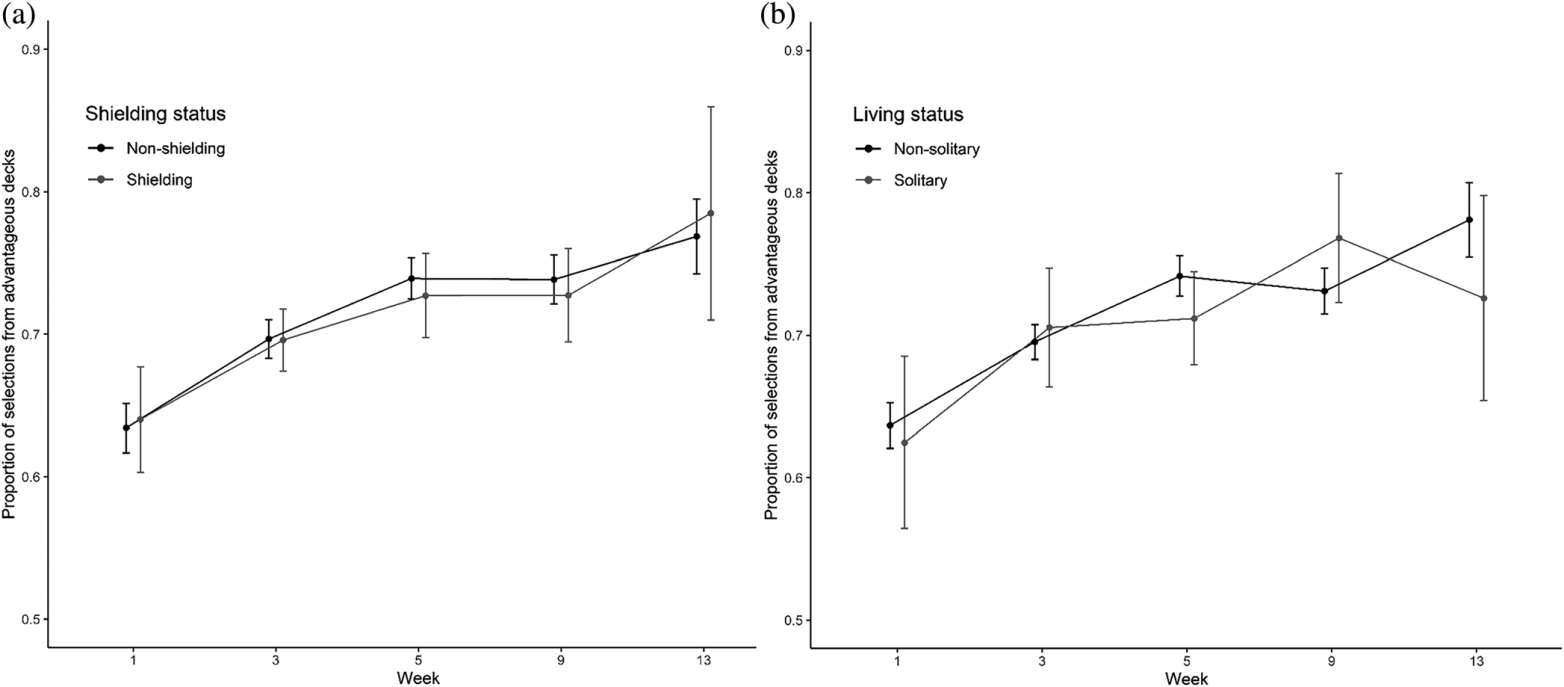
Iowa Gambling task: Mean proportions of selections from advantageous decks for shielding versus non‐shielding (a) and solitary versus non‐solitary participants (b). Error bars show 95% confidence intervals adjusted to remove between‐subjects variance using Morey's (2008) method

Time had a significant effect on the number of advantageous deck selections [*χ* ^2^(4) = 1835.90, *p* < .001; see Figure 1].

Planned contrasts showed improvement (i.e., higher number of advantageous selections) from Week 1 to 3 (*z* = 40.74, *p* < .001), from Week 3 to 5 (*z* = 37.53, *p* < .001), from Week 5 to 9 (*z* = 26.74, *p* < .001), and from Week 9 to 13 (*z* = 19.72, *p* < .001). The subset model showed qualitatively the same results. The shielding status model revealed a significant Group × Time interaction [*χ* ^2^(4) = 11.83, *p* < .05]. This was solely due to a greater improvement from Week 9 to 13 for shielding [*χ* ^2^(1) = 27.72, *p* < .001] than non‐shielding participants [*χ* ^2^(1) = 34.84, *p* < .001]. The living status model also revealed a significant Group × Time interaction [*χ* ^2^(4) = 79.55, *p* < .001]. This was due to a greater improvement from Week 1 to 3 for solitary [*χ* ^2^(1) = 75.70, *p* < .001] than non‐solitary participants [*χ* ^2^(1) = 290.92, *p* < .001], an improvement from Week 3 to 5 for non‐solitary [*χ* ^2^(1) = 221.44, *p* < .001] but not solitary participants [*χ* ^2^(1) = 0.13, *p* = .71], an improvement from Weeks 5 to 9 for solitary [*χ* ^2^(1) = 29.95, *p* < .001] but not non‐solitary participants [*χ* ^2^(1) = 0.26, *p* = .61], and an improvement from Week 9 to 13 for non‐solitary participants [*χ* ^2^(1) = 112.93, *p* < .001] but a deterioration for solitary participants [*χ* ^2^(1) = 36.00, *p* < .001].

### 3.2 Flanker task

**FIGURE 2:**
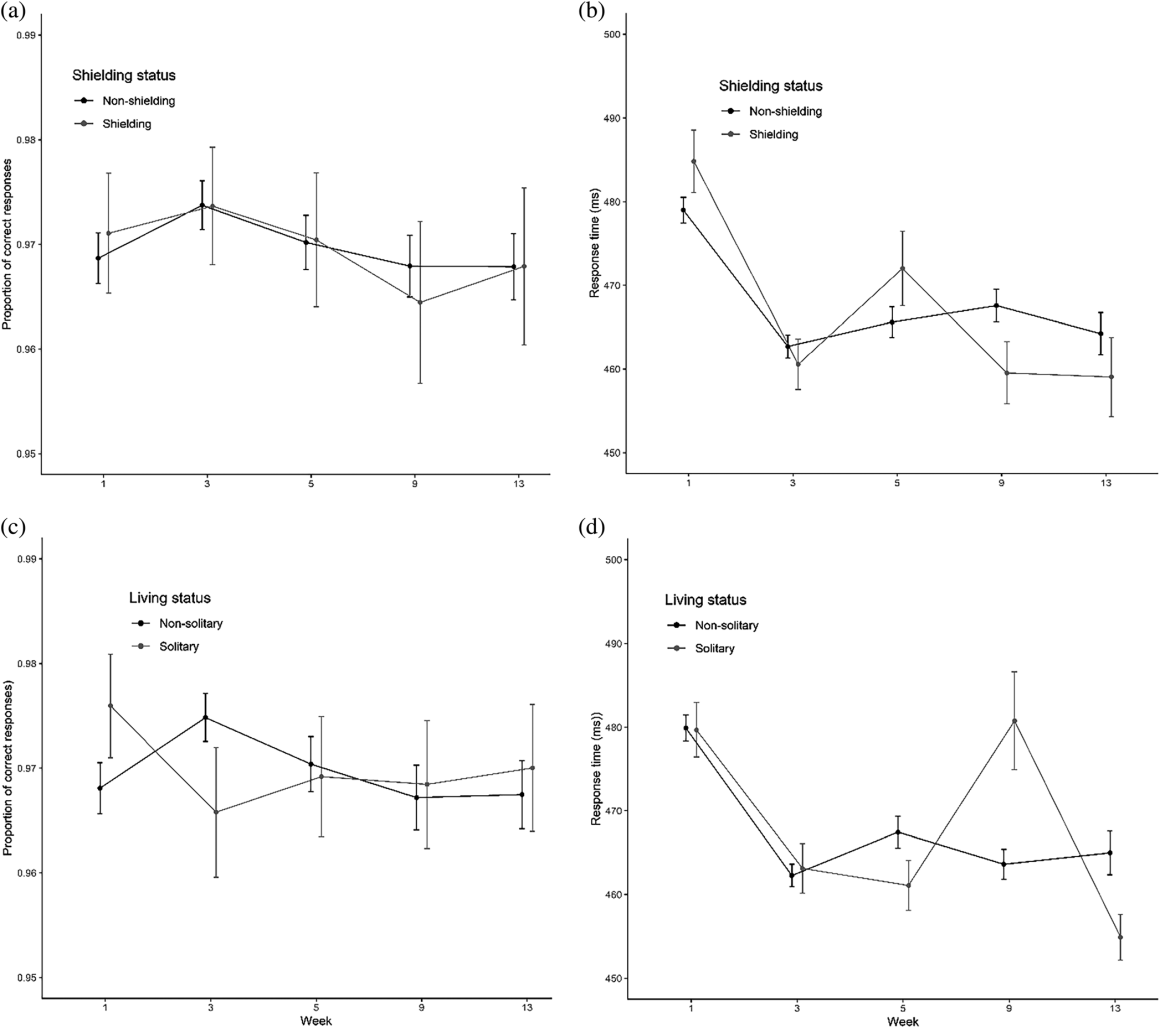
Flanker task: Mean proportions of correct responses and RTs for shielding versus non‐shielding (a and b) and solitary versus non‐solitary participants (c and d). Error bars show 95% confidence intervals adjusted to remove between‐subjects variance using Morey's (2008) method

Time had a significant effect on the number of correct responses [*χ* ^2^(4) = 24.19, *p* < .001; see Figure 2].

Planned contrasts showed no differences between Week 1 and 3 (*z* = 0.80, *p* = .43), a deterioration from Week 3 to 5 (*z* = −2.98, *p* < .01), a further deterioration from Week 5 to 9 (*z* = −3.36, *p* < .001), and no differences between Week 9 and 13 (*z* = −1.80, *p* = .072). The subset model showed qualitatively the same results. We could not test the Group × Time interaction in the shielding status model due to model non‐convergence. The living status model revealed a significant Group × Time interaction [*χ* ^2^(4) = 18.08, *p* < .01]. This was solely due to an improvement from Week 1 to 3 for non‐solitary participants [*χ* ^2^(1) = 23.54, *p* < .001] but a deterioration for solitary participants [*χ* ^2^(1) = 8.47, *p* < .01].

Time also had a significant effect on RT [*χ* ^2^(4) = 453.02, *p* < .001; see Figure 2]. Planned contrasts showed speeding‐up from Week 1 to 3 (*t* = −20.52, *p* < .001), slowing from Week 3 to 5 (*t* = −9.69, *p* < .001), speeding‐up from Week 5 to 9 (*t* = −7.31, *p* < .001) and from Week 9 to 13 (*t* = −6.26, *p* < .001). The subset model showed qualitatively the same results. The shielding status model revealed a significant Group × Time interaction [*χ* ^2^(4) = 41.84, *p* < .001]. This was due to a greater speeding‐up from Week 1 to 3 for shielding [*χ* ^2^(1) = 134.73, *p* < .001] than non‐shielding participants [*χ* ^2^(1) = 332.40, *p* < .001], a greater slowing from Week 3 to 5 for shielding [*χ* ^2^(1) = 31.15, *p* < .001] than non‐shielding participants [*χ* ^2^(1) = 4.79, *p* < .05], and a greater speeding‐up from Week 5 to 9 for shielding [*χ* ^2^(1) = 27.22, *p* < .001] than non‐shielding participants [*χ* ^2^(1) = 3.71, *p* = .054]. The living status model also revealed a significant Group × Time interaction [*χ* ^2^(4) = 88.49, *p* < .001]. This was due to a significant slowing from Week 3 to 5 for non‐solitary [*χ* ^2^(1) = 21.94, *p* < .001] but not solitary participants [*χ* ^2^(1) = 0.20, *p* = .65], a speeding‐up from Week 5 to 9 for non‐solitary participants [*χ* ^2^(1) = 11.87, *p* < .001] but a slowing for solitary participants [*χ* ^2^(1) = 53.78, *p* < .001], and a significant speeding‐up from Week 9 to 13 for solitary [*χ* ^2^(1) = 38.30, *p* < .001] but not non‐solitary participants [*χ* ^2^(1) = 3.37, *p* = .07]. Similar results were obtained when Trial (congruent, incongruent) was included as an additional fixed effect.

### 3.3 Symbol‐learning task

**FIGURE 3:**
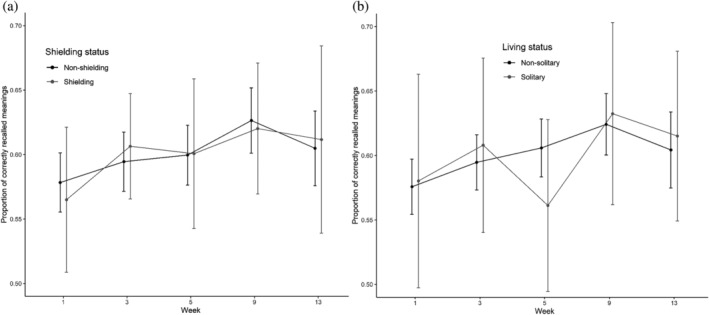
Symbol learning task: Mean proportions of correctly recalled meanings for shielding versus non‐shielding (a) and solitary versus non‐solitary participants (b). Error bars show 95% confidence intervals adjusted to remove between‐subjects variance using Morey's (2008) method

Time had a significant effect on the number of correctly recalled meanings [*χ* ^2^(4) = 25.32, *p* < .001; see Figure 3].

Planned contrasts showed an improvement from Week 1 to 3 (*z* = 4.03, *p* < .001), from Week 3 to 5 (*z* = 4.12, *p* < .001), from Week 5 to 9 (*z* = 3.94, *p* < .001), and a non‐significant deterioration from Week 9 to 13 (*z* = 1.04, *p* = .30). The subset model showed similar results, except there was no difference between Week 1 and 3 (*z* = 1.49, *p* = .14). The Group × Time interaction was non‐significant in both the shielding status [*χ* ^2^(4) = 1.27, *p* = .87] and living status models [*χ* ^2^(4) = 5.12, *p* = .28].

### 3.4 Time production task

**FIGURE 4:**
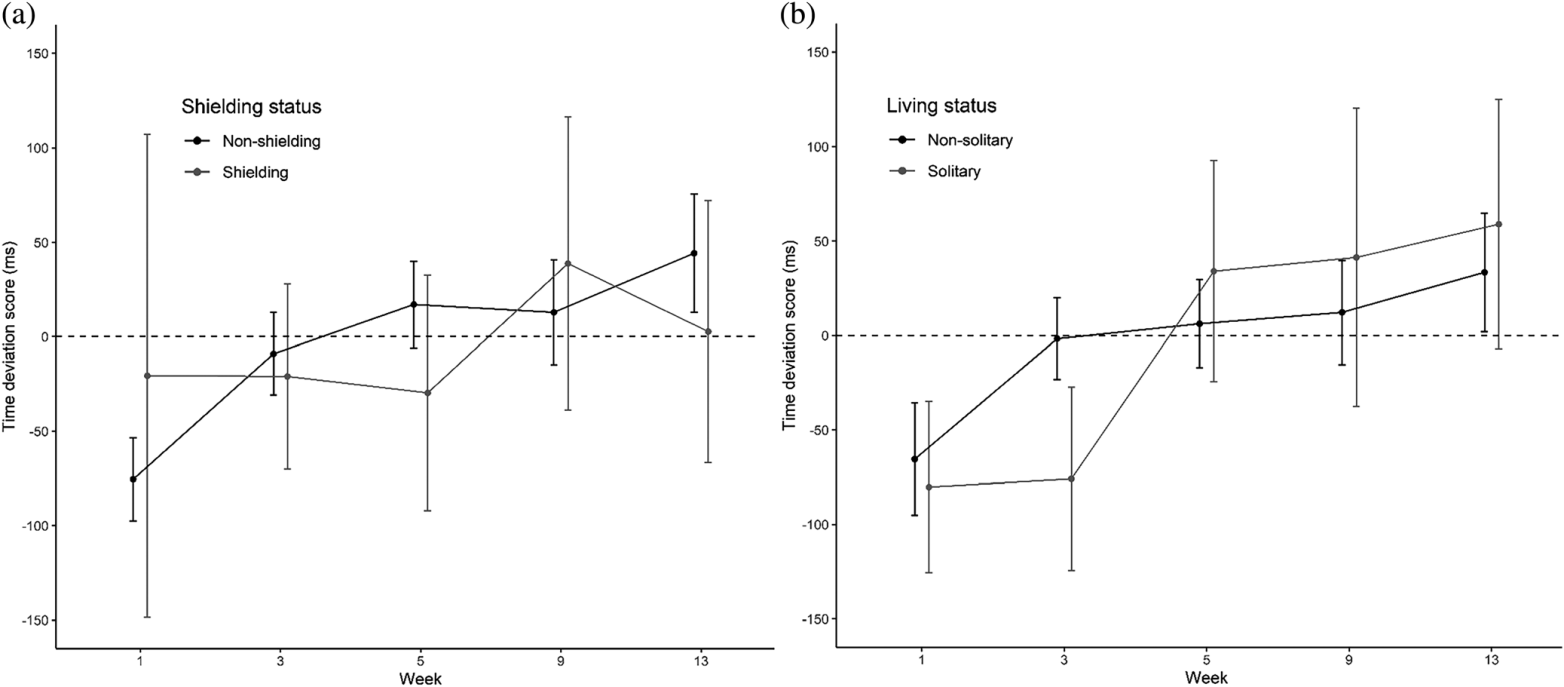
Time production task: Mean time deviation scores (RT—target duration) for shielding versus non‐shielding (a) and solitary versus non‐solitary participants (B). Scores below the dashed line represent underestimation, whereas those above represent overestimation. Error bars show 95% confidence intervals adjusted to remove between‐subjects variance using Morey's (2008) method

Time had a significant effect on time deviation score, or the numerical difference between participants' RT and the amount of time they were asked to estimate/produce [*χ* ^2^(4) = 58.13, *p* < .001; see Figure 4].

Planned contrasts showed a shift towards less underestimation from Week 1 to 3 (*t* = 7.23, *p* < .001), a shift towards overestimation from Week 3 to 5 (*t* = 6.38, *p* < .001), and greater overestimation from Week 5 to 9 (*t* = 5.06, *p* < .001) and from Week 9 to 13 (*t* = 3.93, *p* < .001). The subset model showed qualitatively the same results. The Group × Time interaction was non‐significant in the shielding status [*χ* ^2^(4) = 8.01, *p* = .09] and living status models [*χ* ^2^(4) = 7.24, *p* = .12].

### 3.5 Digit‐symbol substitution task

**FIGURE 5:**
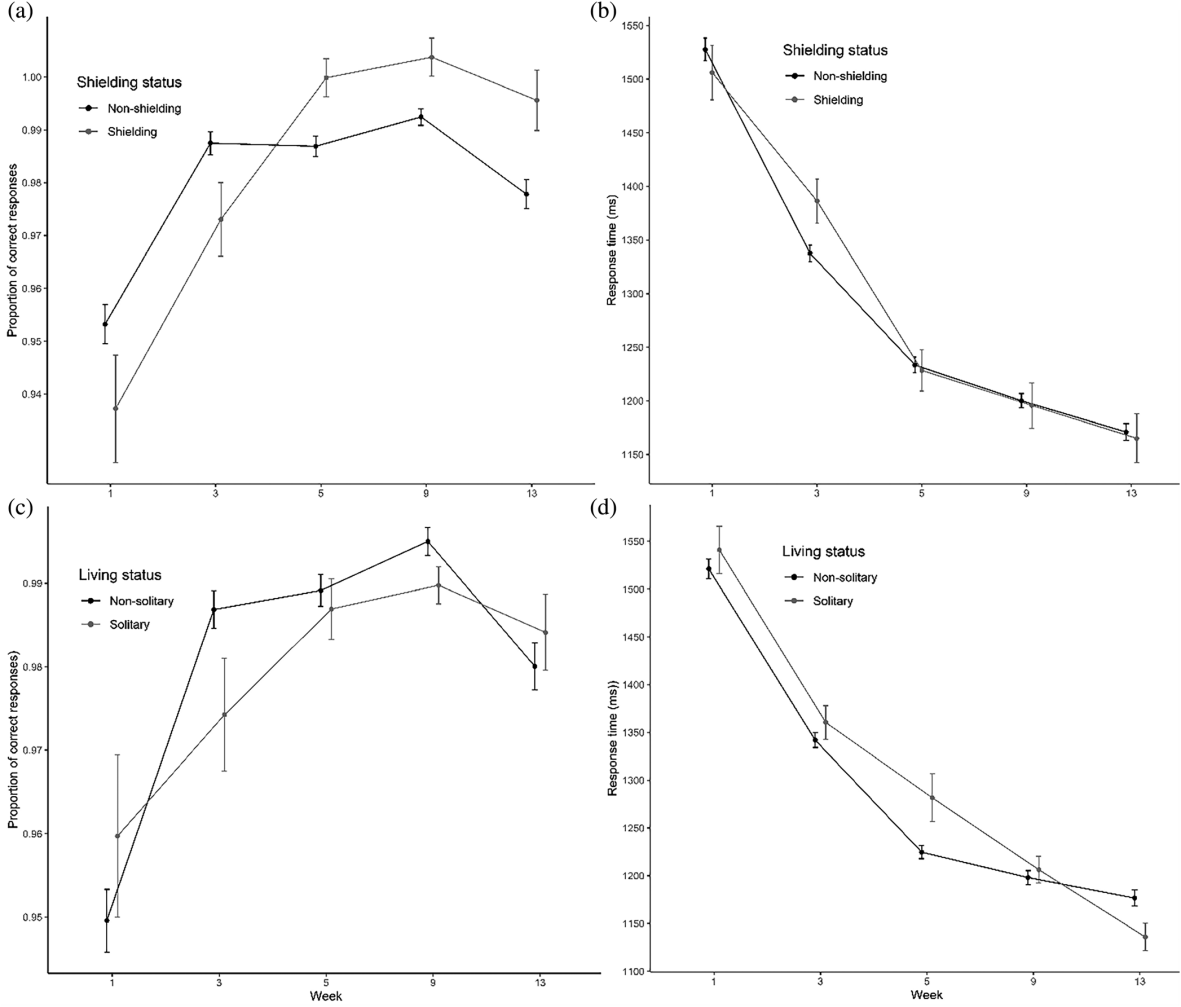
Digit‐symbol substitution task: Mean proportions of correct responses and RTs for shielding versus non‐shielding (a and b) and solitary versus non‐solitary participants (c and d). Error bars show 95% confidence intervals adjusted to remove between‐subjects variance using Morey's (2008) method

Time had a significant effect on the number of correct responses [*χ* ^2^(4) = 980.84, *p* < .001; see Figure 5].

Planned contrasts showed a significant improvement from Week 1 and 3 (*z* = 28.85, *p* < .001), from Week 3 to 5 (*z* = 17.74, *p* < .01), from Week 5 to 9 (*z* = 10.43, *p* < .001), and a deterioration between Week 9 and 13 (*z* = −2.84, *p* < .01). The subset model showed qualitatively the same results. We could not test the Group × Time interaction in the shielding and living status models due to model non‐convergence.

Time also had a significant effect on RT [*χ* ^2^(4) = 7048.80, *p* < .001; see Figure 4]. Planned contrasts showed a speeding‐up from Week 1 to 3 (*t* = −80.31, *p* < .001), from Week 3 to 5 (*t* = −77.13, *p* < .001), from Week 5 to 9 (*t* = −57.40, *p* < .001), and from Week 9 to 13 (*t* = −38.24, *p* < .001). The subset model showed qualitatively the same results. The shielding status model revealed a significant Group × Time interaction [*χ* ^2^(4) = 30.58, *p* < .001]. This was due to a greater speeding‐up from Week 1 to 3 for non‐shielding [*χ* ^2^(1) = 1146.47, *p* < .001] than shielding participants [*χ* ^2^(1) = 87.96, *p* < .001] and a greater speeding‐up from Week 3 to 5 for shielding [*χ* ^2^(1) = 673.61, *p* < .001] than non‐shielding participants [*χ* ^2^(1) = 239.24, *p* < .001]. The living status model also revealed a significant Group × Time interaction [*χ* ^2^(4) = 32.11, *p* < .001]. This was due to a greater speeding‐up from Week 3 to 5 for non‐solitary [*χ* ^2^(1) = 871.16, *p* < .001] than solitary participants [*χ* ^2^(1) = 47.64, *p* < .001], greater speeding‐up from Week 5 to 9 for solitary [*χ* ^2^(1) = 46.28, *p* < .001] than non‐solitary participants [*χ* ^2^(1) = 52.74, *p* < .001], and greater speeding‐up from Week 9 to 13 for solitary [*χ* ^2^(1) = 49.19, *p* < .001] than non‐solitary participants [*χ* ^2^(1) = 22.99, *p* < .001].

### 3.6 Mood rating task

**FIGURE 6:**
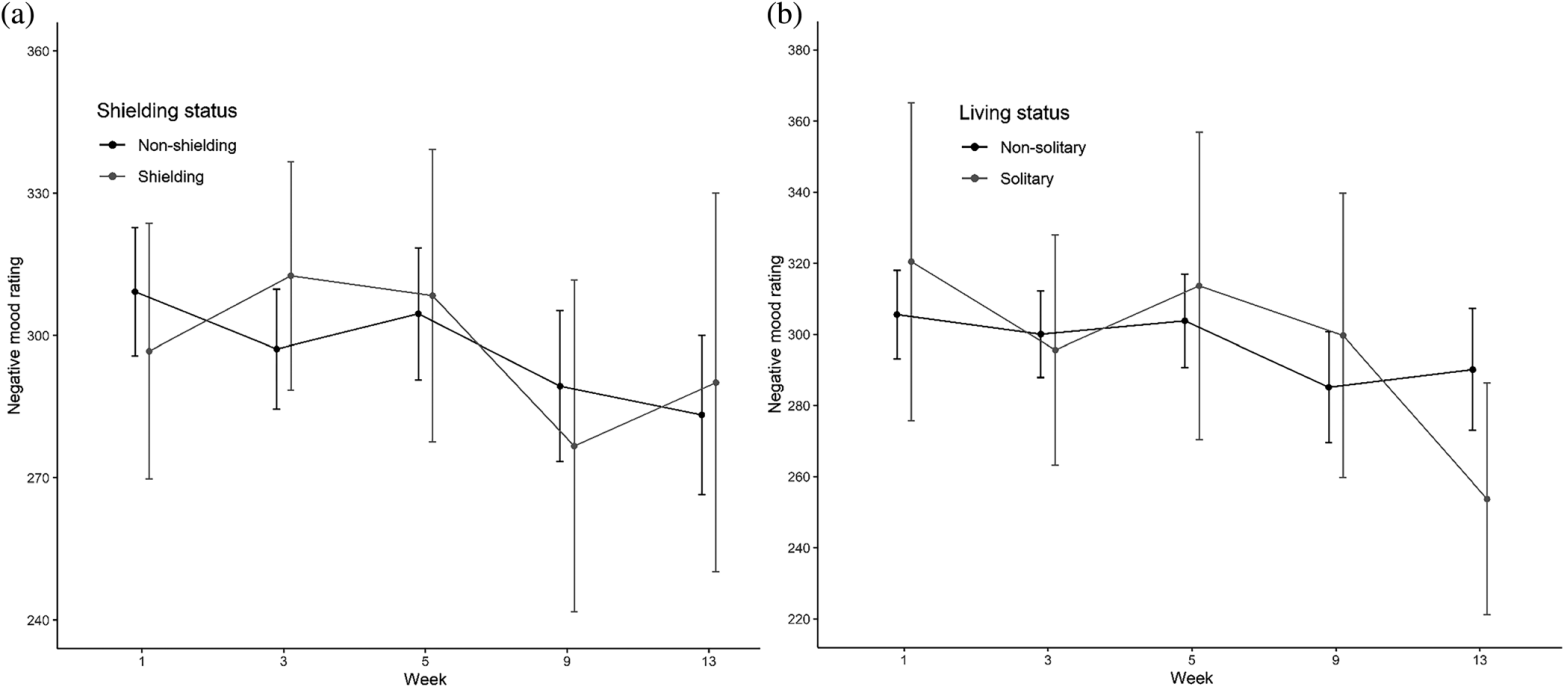
Mood rating task: Mean ratings for shielding versus non‐shielding (a) and solitary versus non‐solitary participants (b). Error bars show 95% confidence intervals adjusted to remove between‐subjects variance using Morey's (2008) method. Higher ratings denote more‐negative mood

Time had a significant effect on NMR [*χ* ^2^(4) = 10.99, *p* < .05; see Figure 6]. Planned contrasts showed an improvement (i.e., lower NMR) from Week 1 to 3 (*t* = −2.28, *p* < .05), a deterioration from Week 3 to 5 (*t* = −2.28, *p* < .05), and an improvement from Week 5 to 9 (*t* = −3.15, *p* < .01) and from Week 9 to 13 (*t* = −2.13, *p* < .05). The effect of Time was marginal in the subset model [*χ* ^2^(4) = 8.94, *p* = .063]. The Group × Time interaction was non‐significant in both the shielding status [*χ* ^2^(4) = 2.14, *p* = 71] and living status models [*χ* ^2^(4) = 4.29, *p* = .37].

## 4 DISCUSSION

Our results suggest that prolonged time in a socially impoverished environment was detrimental to key aspects of cognitive function. Crucially, Group × Time interactions indicated that differential social isolation differentially influenced cognitive function.

We first consider three of our tasks which most‐clearly represent executive function. Iowa Gambling Task (IGT) selections consistently improved as restrictions were eased, except for shielding participants. Shielding participant did not show IGT improvement until between Week 9 and 13 when shielding was ‘paused’ (shielding individuals were required to follow the same restrictions as other individuals during the pause). Flanker task RT performance generally improved as restrictions were eased, with a decline in Week 5 corresponding with an increase in negative mood. Digit‐symbol substitution showed general improvement over time; these improvements were greatest for solitary participants in later weeks, reflecting the broadest re‐opening of society between Week 5 and 9. These solitary participants could now benefit from visiting other people (and having visitors) inside their homes, as well as the re‐opening of many cultural amenities. We additionally tested participants' time‐estimation and symbol‐learning performance. The most‐robust finding for time production was that of a qualitative and quantitative difference in time‐estimation as lockdown conditions eased, from significant underestimation to significant overestimation. Symbol‐learning showed consistent improvement, but no significant Group × Time interactions for either shielding, non‐shielding/solitary, non‐solitary dwellers.

Older adults experiencing cognitive decline show riskier decision‐making in comparison to healthy controls (Smart & Krawitz, 2015); age‐related decline in cognitive processing may lead to decision‐making deficits as adults age (Beitz et al., 2014). Studies using rodents demonstrate direct effects of isolation on decision‐making ability using an adapted version of the IGT (Zeeb et al., 2012). Our IGT analyses show that decision‐making ability improved in less‐restrictive conditions; this was qualified by an interaction with shielding status. This suggests that restricting social behaviours due to the COVID‐19 pandemic led to poorer, riskier decision‐making.

Flanker tasks probe selective attention; we observed flanker RT deficit during the greatest level of isolation. This executive function task‐decrement during severe social restriction is supported by studies involving both older and younger adults (Baumeister & DeWall, 2005; Cacioppo et al., 2000; Twenge et al., 2001). In one study the mere suggestion of a future spent alone led to problems with higher‐order cognitive and self‐regulatory processes (Baumeister et al., 2005); and so the effect of prolonged time spent in a highly restricted social environment is reflected in the poorer performance on the flanker task, particularly at the first timepoint. In addition, fluctuations in flanker task performance corresponding with negative mood rating in our analyses align with previous research indicating an effect of depression on selective attention (see Chepenik et al., 2007 for a review).

Both accuracy and RT data from the digit‐symbol substitution task (DSST) support the hypothesis that cognitive function would be poorer during severe social restrictions. Whilst research involving space exploration has shown minimal effects using the DSST, these trips generally lasted less than a week and involved highly trained participants (Kelly et al., 2005). A decline in cognitive functioning has been linked to prolonged social isolation in older adults (Evans, Martyr, et al., 2019). Research using the Symbol Digit Modality Test (SDMT; Smith, 2007) have shown that information processing and working memory components (similar to those assessed by the DSST) have a reduced rate of decline when older adults maintain social networks and social engagements (Barnes et al., 2004).

Time perception task analyses demonstrate an interesting effect. Rather than improve as lockdown conditions eased, participants evolved from underestimating time‐elapsed when restrictions were severe to overestimating time‐elapsed when restrictions were most relaxed. This suggests that participants' time‐estimation had slowed‐down as restrictions were eased. This result reflects early work on cognitive processing in space expeditions (Ratino et al., 1988). Astronauts' time‐estimation was impaired; particularly, in over‐estimating brief time intervals (2 s) near the end of journey and immediately after landing. This was attributed to astronauts' high workload at the end of a mission. However, the greatest difference was observed in the first time‐estimation assessment immediately after landing on Earth. It is possible that this effect arose from the relaxation or relief associated with successful mission‐accomplishment; this explanation could also apply to the present results. As lockdown restrictions eased, participants felt more relaxed (as evidenced by lower NMR) and began to perceive time passing more slowly.

Significant improvement in negative mood rating as lockdown restrictions eased indicated the benefits of socialisation and freedom of movement. These results support those of Ingram et al. (2020). Cognitive function, particularly attention, varies with mood in isolated (polar) conditions, however these changes were previously considered to be linked to temperature‐related hormone changes (Reed et al., 2001). Our results have implications for research on cognitive ageing, particularly in relation to cognitive reserve.

We have demonstrated that even relatively short‐term social isolation—specifically, reduced social contact with those outside the household—has a negative impact on cognitive abilities/executive functions. These results are in line with studies which demonstrated a link between social isolation and cognitive decline in older adults (Evans, Martyr, et al., 2019; Kuiper et al., 2016). Social interactions are thought to preserve cognitive abilities through the process of cognitive reserve (Stern, 2009); however, in traditional ageing research, it is difficult to differentiate between decline caused by lack of social contact and reduced social contact due to age‐related decline (Gow et al., 2007). The imposed reduction in social contact for our participants (*M*_age_ = 32.1 years, *SD*_age_ = 11.2) allows us to attribute poorer cognitive function to social isolation, as opposed to the reverse. Fluctuations in performance on tasks are also found when comparing participants who lived alone (12% of sample) during ‘lockdown’ to those who lived with others. Specifically, improvements for participants living alone were seen between Week 5 and Week 9, which is when those who were living alone could form ‘extended households’ so that they could visit one other household and be visited by that same other household. Studies of older adults have shown conflicting results with respect to the independent influences of living alone and social isolation on cognitive function (see Evans et al., 2019 for a detailed discussion). Our results support a reduction in cognitive ability for those living alone; however, note that this is a small sample size, and these participants had no opportunity to engage in face‐to‐face social contact to mitigate the increased social isolation experienced whilst living alone during ‘lockdown’.

Another factor which may relate to our results is that of constriction of life‐space. Life‐space refers to the daily extent of movement throughout the environment; that is, a physical measure of spaces (e.g., home, neighbourhood, town, etc.) that a person frequents. Restricted life‐space is linked to increased risk of Alzheimer's dementia (AD) and milder cognitive impairment in older adults (James et al., 2011). Life‐space‐constrained participants—for instance, those who rarely left their home or neighbourhood—were twice as likely to develop AD than those with larger life‐space, controlling for social network size (James et al., 2011). These results and our own suggest that physically restrictive conditions can drive cognitive decline, as opposed to only social restrictions/social isolation. Therefore, strategies to alleviate cognitive decline should not focus exclusively on encouraging online social interaction, as this does not expand life‐space.

Restrictions to, or reduced, physical activity may also be linked with reduced cognitive ability. Physical activity has been shown to protect against dementia and benefit cognition (Fratiglioni et al., 2004). Whilst engaging in aerobic exercise seems to improve older adults' abilities on tasks involving executive control (Kramer et al., 1999), it is difficult in research involving older adults to unpick the relationship between cognitive decline, social interaction, and physical activity (Richards et al., 2003), or between physical function (e.g., mobility), life‐space, and cognitive ability (De Silva et al., 2019). It is a limitation of the current study that physical activity was not tracked across timepoints. However, at timepoint 1, 52.7% of participants reported having increased or perceived no‐change to their level of physical activity since the ‘lockdown’ conditions were imposed. Therefore, a decrease in physical activity due to restrictions cannot account for the decline in cognitive function within this group. These reported changes in physical activity support the conclusion that reduced social interaction, and life‐space, account for our results, with easing of restrictions leading to graded improvement in performance on cognitive tasks.

Our study is somewhat limited as, due to the immediate instigation of lockdown measures within the UK, we were unable to gather baseline measures of cognitive function. As a consequence, it is not possible to show the level of initial cognitive decline, or any adaptation of cognitive processes to the socially impoverished conditions. However, the finding of improvements over time seen across tasks supports theories of cognitive enrichment and plasticity (Hertzog et al., 2009). Our results demonstrate plasticity of cognitive function, with graded improvement in tasks as restricted eased qualified by differing patterns across groups. The effect of practice on task improvements cannot be ruled out without baseline measures. It is important to note though that the differing patterns of improvement for the shielding and living alone participants, which correspond with differing changes to restrictions aligned with these groups, suggest this is not the case. Similarly, fluctuations in improvements linked to mood further indicate that the observed results are driven by the very nature of restrictions, rather than repeated testing within our study.

We demonstrate that restrictive living conditions consequent of the COVID‐19 pandemic related to poorer cognitive performance. Easing of restrictions allowed more mobility, and social contact coincided with improvement in a number of tests of cognitive function. This pattern was reinforced by evidence that individuals who were more isolated (shielding participants) demonstrated longer‐lasting deficits in cognition. Our results support the theory of cognitive reserve and suggest that maintaining social relationships throughout the lifespan plays a role in maintaining cognitive ability. Continued restrictions to social contact and life‐space may be highly detrimental to cognitive function. As such, if lockdown conditions continue to be used in the fight against the COVID‐19 pandemic, strategies to alleviate cognitive decline during prolonged restrictive conditions should be considered. As a true substitute for social contact and life‐space is unlikely to be found, policymakers may wish to also consider the effect on cognitive function when implementing restrictions. Future research may wish to address longer‐term effects on cognitive function as restrictions continue to be relaxed and then tightened.

## CONFLICT OF INTEREST

We confirm that none of the authors have a conflict of interest.

## Supporting information


**Appendix** **S1.** Supporting Information.

## Data Availability

The data and scripts that support the findings of this study are openly available in OSF at https://osf.io/kvpbt/.

## Endnote

^1^
Mixed‐effects models typically fail to converge when testing too many or too complex effects that are not supported by the underlying data (see Bates et al., 2015), especially when modelling binary outcome variables.
