# Supplementary material for: Social isolation during COVID‐19 lockdown impairs cognitive function
Source: Appl Cogn Psychol. 2021 Mar 24;35(4):935–47. doi: 10.1002/acp.3821 (PMC8250848; doi:10.1002/acp.3821)
Supplement: Supplementary file 1 — Appendix S1. Supporting Information. [file ACP-35-935-s001.docx]

**Descriptions of measures**

**Iowa gambling task**

In this task (adapted from Bechara et al., 1994), participants selected cards from one of four decks of cards to win as much ‘game money’ as possible. Participants began the task with £2000, and were told that each deck held cards that would either reward them or penalise them. There were 100 randomised trials with no time limit, half of which involved a penalty. There were two ‘advantageous’ decks that resulted in an overall gain in the long run (£50 reward, £50 penalty), and two ‘disadvantageous’ decks that penalised participants the most (£100 reward, £250 penalty). The number of selections from the advantageous decks served as a measure of decision-making.

**Flanker task**

In this task (adapted from Wylie et al., 2007), participants were presented with a target arrow located in the centre of the screen and two distractor arrows (i.e., flankers) located on each side of the target arrow. Participants were told to ignore flankers and press the J button when the target arrow pointed right, or the F button when it pointed left (instructed to respond as fast and accurately as possible). On congruent trials, flankers pointed in the same direction as the target arrow. On incongruent trials, flankers pointed in the opposite direction. Trials began with a 500msec fixation cross. The target arrow and flankers were displayed until participants responded, and were followed by a 750msec inter-trial interval. There were 20 practice trials with feedback on accuracy and 80 experimental trials, both of which were presented in random order and had equal numbers of the four direction-by-congruency combinations. The number of correct responses and RTs (for correct responses) served as a measure of selective attention, or the ability to inhibit task-irrelevant flankers.

**Symbol learning task**

In this task (adapted from Yang et al., 2017), participants studied and then recalled the meanings of Mandarin characters. We used 50 visually-simple characters with concrete meanings (e.g., ‘日’ denoting ‘sun’). The characters were randomly split into five lists, one for each timepoint. The lists were counterbalanced across participants and timepoints, and the characters were presented in random order both in the learning and the recall phase. In the learning phase, participants had unlimited time to study a character-translation pair and pressed the space bar to move onto another pair. After the learning trials, participants completed a short distractor task, in which they solved 10 mathematical problems that required adding or subtracting two-digit numbers (e.g., 69 + 63 = ?). The problems were presented in random order, one at a time. In the recall phase, participants were presented with a character and had unlimited time to recall and type in its meaning (or the word ‘nothing’ if they could not recall any information). The number of correctly recalled meanings served as a measure of learning ability.

**Digit-symbol substitution task**

In this task (adapted from Chatterjee et al., 2019, Version 1), participants were presented with an array of nine digit-symbol pairs (i.e., lookup table) at the top of the screen and a target digit-symbol pair in the centre of the screen. Participants pressed the J button if the target pair matched with either of the nine pairs in the lookup table, or the F button if it did not (as fast and accurately as possible). The target pair and lookup table were displayed until participants’ response, and were followed by a 300msec inter-trial interval. There were 10 practice trials with feedback on accuracy and 50 experimental trials involving a different set of symbols, both of which were presented in random order. Note that we used different target pairs and the same lookup table at each timepoint. This resulted in varying ratios of matching to mismatching trials across the timepoints. The number of correct responses and RTs (for correct responses) served as a measure of working memory.

**Time production task**

In this task (adapted from Tortello et al., 2020), participants estimated when a certain amount of time (500, 1,000, 1,500, or 4,000msec) had passed. Trials began with instructions on the target duration (e.g., ‘produce 1,000msec’) that were displayed for 5,000msec. A response cue (‘?’) was then displayed until participants pressed the space bar to indicate when the given amount of time had passed. There was a 1,000msec intertrial interval. There were four practice and 16 experimental trials, both of which were presented in random order and involved equal numbers of the four target durations. Time deviation (in msec) was calculated by subtracting the target duration from the response time, and served as a measure of time estimation (negative values indicated underestimation and positive values indicated overestimation).

**Mood rating task**

We used 10 negative items from Grove and Prapavessis’ (1992) abbreviated Profile of Mood State (POMS) scale, with two items taken from each of the five subscales – Confusion (‘forgetful’, ‘unable to concentrate’), Tension (‘anxious’, ‘uneasy’), Depression (‘helpless’, ‘sad’), Fatigue (‘exhausted’, ‘worn out’), and Anger (‘angry’, ‘annoyed’). Participants rated their mood on each of the 10 items at that point in time using a 100-point slider scale (where higher ratings denoted greater negative mood). Ratings summed across all the items served as a measure of negative mood. At Timepoint 1 (Week 1), Cronbach’s α for the entire (negative) scale was .91, and between .69 and .89 for the five subscales (*M*=.85).

**References**

Bechara, A., Damasio A.R., Damasio H., Anderson S.W. (1994). Insensitivity to future consequences following damage to human prefrontal cortex. Cognition, 50(1-3), 7-15. <https://doi.org/10.1016/0010-0277(94)90018-3>

Chatterjee, D., Gavas, R. D., Chakravarty, K., Sinha, A., & Lahiri, U., (2019). Evaluating age-related variations of gaze behavior for a novel digitized-digit symbol substitution test. Journal of Eye Movement Research, 12(1):5. <https://doi.org/10.16910/jemr.12.1.5>

Grove, J. R., & Prapavessis, H. (1992). Preliminary evidence for the reliability and validity of an abbreviated Profile of Mood States. International Journal of Sport Psychology, 23(2), 93–109.

Tortello, C., Agostino, P. V., Folgueira, A., Barbarito, M., Cuiuli, J. M., Coll, M., Golombek, D. A., Plano, S. A., & Vigo, D. E. (2020). Subjective time estimation in Antarctica: The impact of extreme environments and isolation on a time production task. Neuroscience Letters, 23, 725:134893. <https://doi.org/10.1016/j.neulet.2020.134893>

Wylie, S. A., Ridderinkhof, K. R., Eckerle, M. K., & Manning, C. A. (2007). Inefficient response inhibition in individuals with mild cognitive impairment. Neuropsychologica, 45(7), 1408-1419. <https://doi.org/10.1016/j.neuropsychologia.2006.11.003>

Yang, C., Potts, R., & Shanks, D. R. (2017). The forward testing effect on self-regulated study time allocation and metamemory. Journal of Experimental Psychology: Applied, 23(3), 263-277. <https://doi.org10.1037/xap0000122>
